# Systemic Inflammation in Duchenne Muscular Dystrophy: Association with Muscle Function and Nutritional Status

**DOI:** 10.1155/2015/891972

**Published:** 2015-08-24

**Authors:** Oriana del Rocío Cruz-Guzmán, Maricela Rodríguez-Cruz, Rosa Elena Escobar Cedillo

**Affiliations:** ^1^Laboratorio de Biología Molecular, Unidad de Investigación Médica en Nutrición, Hospital de Pediatría, Centro Médico Nacional Siglo XXI, IMSS, 4th Floor, Avenida Cuauhtémoc No. 330, Colonia Doctores, Delegación Cuauhtémoc, 06725 México, DF, Mexico; ^2^Servicio de Electrodiagnóstico y Distrofia Muscular, Instituto Nacional de la Rehabilitación, México, DF, Mexico

## Abstract

Inflammation described in patients with Duchenne muscular dystrophy (DMD) may be related to loss of muscle function or to obesity. It is unknown if circulating proinflammatory cytokines (IL-6, IL-1, and TNF-*α*) levels are associated with muscle function. The purpose was to evaluate whether an association exists between systemic inflammation with muscle function and nutritional status in DMD patients. In 66 DMD patients without corticosteroid treatment, the following were evaluated in serum: cytokines (IL-1, IL-6, and TNF-*α*), C-reactive protein (CRP), leptin, adiponectin, and creatine kinase (CK). Muscle function was evaluated using Vignos Scale. Patients with better muscle function had the highest concentration of CK, IL-1, and TNF-*α* compared with less muscle function. No differences in IL-6 and adiponectin concentration were identified among groups with different levels of muscle function. Also, no differences were observed in the concentration of cytokines among groups with different nutritional status levels (underweight, normal weight, and overweight/obese). However, CRP and leptin were increased in the obese group compared with normal and underweight subjects. Systemic inflammation is increased in patients with better muscle function and decreases in DMD patients with poorer muscle function; nevertheless, systemic inflammation is similar among different levels of nutritional status in DMD patients.

## 1. Introduction

Duchenne muscular dystrophy (DMD) is an X-linked muscle disease [1] caused by mutations in the DMD gene that code for dystrophin. This protein has an important structural role during muscle contraction. Fragile muscle fiber and instable sarcolemma are some of the outcomes of the protein mutation. These alterations lead to chronic inflammation, which is an important feature of the disease pathophysiology. First, if the fragile muscle suffers a stretch process, the sarcolemma experiences a mechanical stress. Consequently, Ca^2+^ levels increase and activate the necrosis [2]. Next, necrotizing myofibers are attacked and removed principally by M1 macrophages. At the same time, M1 cells secrete proinflammatory cytokines and recruit additional inflammatory cells, increasing the inflammation process. It has been reported that biopsy of the dystrophic muscle shows overexpression of proinflammatory cytokines such as tumor necrosis factor alpha (TNF-*α*), interleukin-1 (IL-1), and interleukin-6 (IL-6) in comparison with healthy muscles, leading to chronic inflammation [3–6]. Because muscle biopsy is an invasive method, there is no information about the inflammation during the progression of muscle damage in DMD patients. Despite this, it has been demonstrated that DMD patients have increased levels of cytokines (TNF-*α*, IL-6) in serum compared with healthy subjects [3, 4, 7]. There are no studies about circulating biomarkers of inflammation throughout evolution of dystrophy.

DMD patients usually present generalized motor delays and gait difficulties in early childhood. Muscle weakness is progressive, causing loss of ambulation by early adolescence (between 9 and 12 years of age) [1]. Some assessment scales, such as the Vignos Scale, have been used to evaluate the motor function of neuromuscular diseases. The Vignos Scale provides information about the degree of functional dependence of the patient and measures lower extremity function [8]. To support this assessment, clinical circulating biomarkers such as creatine kinase (CK) are used where high levels of CK reflect muscle damage. However, it is important to consider that CK levels are not reliable because they vary considerably under several independent stress conditions, not necessarily associated with muscle damage [9].

Additionally, the inflammation process might exhaust the degeneration and regeneration cycles of the muscle fiber, provoking the replacement of muscle fibers by connective and adipose tissue [10]. Adipose tissue accumulated in muscle tissue may lead to metabolic alterations such as obesity. It is well known that DMD patients develop obesity from the age of 7 years [11] not only due to glucocorticoid treatment as we previously reported. A sample of DMD patients (*n* = 66) who were not taking medication, including glucocorticoids, demonstrated 18.3% underweight, 22.7% overweight, and 68% obesity [12]. It is well known that obesity is associated with low-grade inflammation of white adipose tissue due to the secretion of proinflammatory cytokines such as TNF-*α*, IL-1, and IL-6 [13–15]. In the same way, C-reactive protein (CRP) is also increased in the serum of obese subjects. Although CRP is not a cytokine, it is a useful inflammatory marker for obesity [15, 16].

Other molecules associated with obesity are leptin and adiponectin. Leptin has been demonstrated to be increased in obese children, whereas adiponectin is decreased. Also, leptin has a positive relationship with CRP and is related to the amount of body fat [16].

Chronic inflammation caused by muscle damage or due to obesity in DMD patients has an important impact on disease progression [10]. Identification of inflammatory status during the progression of the dystrophy is clinically important because it will be useful for the detection of the degree of muscle damage, increasing the possibility to propose early interventions regardless of the origin of the inflammation, whether muscle damage or obesity. It has an important impact on the progress of the clinical characteristics in DMD patients.

Thus, the purpose of this investigation was to evaluate whether an association exists among systemic inflammation with muscle function and nutritional status in DMD patients.

## 2. Materials and Methods

### 2.1. Subjects and Methods

The study was conducted at the Laboratory of Molecular Biology, Medical Research Unit in Nutrition, Pediatric Hospital, Centro Medico Nacional Siglo XXI (CMN SXXI), Instituto Mexicano del Seguro Social (IMSS). All DMD patients seen at the outpatient Electrodiagnostic Muscular Dystrophy Service at the National Institute of Rehabilitation were recruited for the cross-sectional study between January 2011 and December 2013. Subjects with a clinical diagnosis of DMD were candidates to participate in the study and confirmatory molecular diagnosis of dystrophy was carried out as described in a recent report in the same patients [12]. Therefore, 66 patients (aged 4–18 years) with confirmed diagnosis of dystrophy were included in the present study. Children were not included if they received therapy with corticosteroids. None of the patients was taking medications during the study.

The study was approved by the institutional ethics committee of the IMSS. Parents and patients received an explanation of study fundamentals, procedures, benefits, right to confidentiality, and right to withdraw from the study if they wished. All parents provided written informed consent in adherence with the human subjects' guidelines of the institutional ethics committee.

After a 12 h overnight fast, peripheral blood was collected from each patient in a Vacutainer without anticoagulant to quantify the cytokines TNF-*α*, IL-6, IL-1, CRP, and CK. Serum samples were kept at −70°C until analysis. A medical history was then obtained by trained personnel using the Vignos Scale. Finally, anthropometric measurements such as weight, height, and body composition by dual-energy X-ray absorptiometry (DEXA) were measured.

### 2.2. Vignos Scale Measurement

There are different procedures to evaluate muscle function in DMD patients. One of the most utilized is the Vignos Scale, which measures lower extremity function. It is painless and easy to assess and completion time is short. The range of the scale has been classified from 1 to 10 as follows [19]:

1. walks and climbs stairs without assistance,
2. walks and climbs stairs with aid of railings,
3. walks and climbs stairs slowly with aid of railings (>25 sec for seven standard steps),
4. walks unassisted and rises from chair but cannot climb stairs,
5. walks unassisted but cannot rise from chair or climb stairs,
6. walks only with assistance or walks independently with long leg braces,
7. walks in long leg braces but requires assistance for balance,
8. stands in long leg braces but unable to walk even with assistance,
9. confined to wheelchair,
10. confined to bed.

Based on the chronological functional stages, patients were classified into three groups according to the Vignos Scale: Group A that includes patients with* independent ambulation* (range 1–5); Group B that includes patients with* assisted ambulation* (range 6–8); and Group C that includes patients with* wheelchair*-*limited mobility *(range 9-10) [20].

### 2.3. Anthropometric Measurements

Body weight (kg) and height (m) measurements were all performed by trained personnel. For subjects who were able to stand erect, height was measured with a wall-mounted stadiometer (Model 208, Seca). For subjects unable to stand erect, length was measured on a flat table with the subject supine. Weight of ambulatory patients was measured using a Tanita digital scale (Model BWB-700) and for wheelchair-bound patients was measured using a wheelchair scale (Model 954) with the subject wearing light clothing and without shoes.

In this study, patients were classified according to their nutritional status. Diagnosis of overweight and obesity was obtained using body mass index (BMI) expressed as percentiles. Children with BMI ≤ 5th percentile were classified as underweight; those with BMI > 5th but <85th percentile as normal weight; BMI ≥ 85th but <95th percentile as overweight; and BMI ≥ 95th percentile as obese, according to criteria established by the Centers for Disease Control and Prevention (CDC) in 2009 in regard to BMI for children and teenagers (http://www.cdc.gov/healthyweight/assessing/bmi/childrens_bmi/about_childrens_bmi.html).

### 2.4. Body Composition

Body composition measurements were carried out using DEXA (Lunar Prodigy, GE Medical Systems, Madison, WI). enCore software, v. 2004 (Lunar Corporation), was used to analyze whole-body DEXA scans.

### 2.5. Biochemical Assays

CRP and proinflammatory cytokines IL-1, IL-6, and TNF-*α* (pg/mL and mg/dL) were quantitated by a solid-phase chemiluminescent immunometric assay using commercial kits (IMMULITE 1000 Analyzer, DPC, Siemens, Malvern, PA). Leptin (ng/mL) and adiponectin (pg/mL) were measured using a commercial kit (Linco Research, St. Louis, MO) based on radioimmunoanalysis. CK (U/L) was determined by chemiluminescent immunometric assay by a commercial kit (Spinreact, Sant Esteve, Spain).

### 2.6. Statistical Analysis

Statistical analysis was performed using SPSS Statistics software (v. 22.0; SPSS Inc., Chicago, IL). Data are presented as mean ± standard deviation (SD) or median (minimum, maximum) as appropriate. Statistical significance was defined at *P* < 0.05. Distribution of the studied variables was examined using the Shapiro-Wilk test. Analysis of differences among groups was conducted with one-way analysis of variance using Bonferroni's or Dunnet post hoc. For data not normally distributed, nonparametric statistics such as Kruskal-Wallis and Mann-Whitney *U* test to identify differences between groups were used.

## 3. Results

### 3.1. Study Subjects

In this study, 66 patients with clinical and molecular diagnosis of DMD were included.  Table 1 summarizes the clinical, anthropometric and biochemical parameters of DMD patients. In this sample study, patient ages were 9.4 ± 3.1 years (mean ± SD); 18 patients were wheelchair-bound. The mean of Vignos Scale value indicates that patients are capable of walking and climbing stairs but with difficulties. According to the CDC, normal-weight patients have a BMI of 46.3 ± 35.6. On the other hand, as expected, the average concentration of CK (11475.0 ± 6830.5 U/L) is elevated with respect to normal values.

### 3.2. Systemic Inflammation and Muscle Function in DMD

Because chronic inflammation is associated with muscle damage impacting on the progress of Duchenne dystrophy, we decided to classify the study population according to muscle functional status using the Vignos Scale.

As expected, our results show that patients with independent ambulation (Vignos Scale A) (Table 2) had a higher concentration of proinflammatory cytokines IL-1 and TNF-*α* in comparison with wheelchair-limited mobility patients (Vignos Scale A). In the same sense, CK, the biomarker of muscle damage, is also increased in the same group compared with the Vignos Scale A and B groups. In contrast, patients with wheelchair-limited mobility (Vignos Scale C) have a lower concentration of proinflammatory cytokines (IL-1 and TNF-*α*) and CK in comparison with Vignos Scale A group. Nonetheless, no differences in IL-6 were observed among groups.

IL-1 concentration decreased four times in Vignos Scale C group in comparison with Vignos Scale A group and decreased six times in Vignos Scale B group. Regarding TNF-*α*, its concentration decreased by one in Vignos Scale C group compared with Vignos Scale A group. CK concentration was reduced four times in Vignos Scale C group with respect to Vignos Scale A group (Table 2).

Leptin and CRP increased three and five times, respectively, in Vignos Scale C group compared with Vignos Scale A group. In contrast, no differences in adiponectin concentration were detected among groups (Table 2).

Accordingly, patients from Vignos Scale C group have the highest percentage of body fat, body weight, and lean body mass compared with patients with better muscle function (Table 2). Although both lean and fat mass showed a higher increase in Vignos Scale C group than Vignos Scale A group, it does not occur in the same proportion. In Vignos Scale A group, we can observe that the amount of lean body mass (kg) is almost three times higher than body fat mass (kg), but in Vignos Scale C group the proportion between lean and fat body mass (kg) is similar (Table 2).

### 3.3. Systemic Inflammation and Nutritional Status in DMD

We did not observe significant differences in the concentration of proinflammatory cytokines such as IL-1, IL-6, and TNF-*α* among groups with different nutritional status. These results suggest that the inflammatory status produced by these cytokines is similar, independent of the nutritional status of patients. In the same sense, no differences were found in age, concentration of CK, and Vignos Scale among levels of nutritional status. In contrast, as expected, the concentration of CRP in overweight/obese patients was higher than that in the other two groups (Table 3). Accordingly, body weight, body fat mass, lean mass, and leptin increased significantly (*P* < 0.05) in overweight/obese boys in comparison with patients with healthy weight (Table 3). Increase of these parameters in obesity is well known, although no differences in adiponectin concentration among nutritional status groups were observed (Table 3).

## 4. Discussion

Inflammation plays a main role in the development of the pathology in DMD. Although it is well known that chronic and exacerbated inflammation can provoke the loss of muscle regeneration, currently there are insufficient human studies that identify whether an association exists between systemic inflammation with muscle function and nutritional status in DMD patients. The results of this study suggest that systemic inflammation is associated with muscle function in patients with DMD.

Because muscle biopsy is an invasive procedure, there is no information about the inflammation during muscle damage progress of DMD patients. Consequently, in an attempt to identify the inflammation process of dystrophy throughout evolution in a noninvasive manner, we analyzed inflammatory molecules in serum. Although it is well known that the inflammation in DMD is mainly localized in muscle fibers from damaged muscle, the findings of this investigation lead us to consider some cytokines as biomarkers of systemic inflammation that may indicate muscle injury.

### 4.1. Systemic Inflammation and Muscle Function in DMD

Inflammation has an important impact on the physiopathology of DMD, where the immunology cells secrete cytokines that amplified the inflammation process. Exacerbation of inflammation can provoke the loss of muscle regeneration and decrease muscle mass; consequently, there is a loss of muscle function.

Our results show that proinflammatory cytokines (IL-1 and TNF-*α*) are decreased in the serum of patients with less muscle function. It is well known that cytokines are increased in dystrophic patients compared to healthy subjects. For instance, TNF-*α* concentration in serum of DMD patients is increased eight times in comparison with healthy boys (30.2 ± 9.5 versus 3.6 ± 0.9 pg/mL) of the same age (8.1 ± 1.9 years old) [18]. IL-6 is also increased in serum of DMD individuals (3.77 ± 2.71 pg/mL) compared with healthy subjects (1.93 ± 1.38 pg/mL) [7]. Therefore, cytokines may have a significant impact on the inflammatory process. There are some studies that suggest a correlation between the inflammatory cytokine expression and muscle function in DMD. de Pasquale et al. demonstrated a higher expression of proinflammatory cytokine (IL-17) in muscle biopsy from DMD patients with the lower motor outcome evaluated by North Star Ambulatory Assessment score (NSAA), suggesting that IL-17 mRNA levels and functional outcome are negatively associated [6].

According to those findings, CK was also reduced in the same patients. It has been reported that CK decreases in older patients [21, 22]. Saito et al. reported in young patients (<20 years old) that TNF-*α* concentration (60.7 ng/L) was approximately five times higher compared with patients >20 years of age (12 ng/L) [4].

Based on these studies and according to our results, we propose that the concentration of cytokines (serum inflammatory markers) is particularly increased in the early stage of the disease such as in younger DMD patients with independent ambulation. This proposal is also based on other studies [23–25] where the authors described that the number of inflammatory cells decreases in muscle tissue of dystrophic patients after the age of 8 years and the quantity of these cells is greater in the first years of the patient's life. However, it is important to consider that muscle fibers contribute to inflammation due to their ability to produce cytokines as demonstrated in cells of dystrophic and nondystrophic mice. Cell lines of myocytes yield and secrete different cytokines into the medium such as IL-1, IL-6, and TNF-*α* and send signals to the muscle to synthesize additional cytokines [26–28]. Therefore, younger patients with better muscle function have more muscle mass with a higher production of cytokines. The concentration of these cytokines in serum is increased in comparison with patients with less muscle function.

Based on these studies and because we observed that patients with the most affected muscle function (wheelchair-limited mobility) have the lowest concentration of IL-1, TNF-*α*, and CK compared with patients with less muscle damage (independent ambulation), we hypothesized that the decrease in concentration of these cytokines and CK may result from muscle loss in DMD patients. This hypothesis is also supported by Emery and Muntoni who suggest that the declining CK levels in serum of DMD patients is associated with a decrease in ambulatory skills and disease progression [22].

### 4.2. Systemic Inflammation and Nutrition Status in DMD

Cytokines are secreted by adipose tissue and this secretion is increased in obese subjects. The muscle of dystrophic boys is replaced by fat mass. Therefore, inflammation in DMD patients may also result from obesity.

We observed that concentration of inflammatory molecules, Vignos Scale, and age was similar among groups with different levels of nutritional status, suggesting a similar inflammatory status independent of the obesity grade. For instance, underweight and obese children have similar cytokine values. Interestingly, our results suggest that, in underweight patients, systemic inflammation may be caused in part by the pathology of DMD because these patients have less body fat mass. However, in patients with overweight or obesity, this systemic inflammation may be originated by adipose tissue. It has been reported that proinflammatory cytokines are increased in obese children. The authors reported a higher concentration of serum IL-6 (0.14 versus 0.05 pg/mL) and TNF-*α* (9.2 pg/mL versus 8.1) in obese Mexican children (13 years old) than in nonobese children [29].

Others molecules associated with obesity are CRP, adiponectin, and leptin. Liver and adipose tissue produce CRP and both may contribute to elevated plasma CRP levels in obesity. A high amount of body fat may produce an important part of the circulating CRP [17, 30]. As expected, CRP and leptin were increased in overweight and obese boys because body fat was increased. These two molecules (CRP and leptin) were also increased in the Vignos Scale C group, possibly explained because subjects in the Vignos Scale C group had the highest values of fat mass [11].

However, it is important to consider that this study has some limitations. We evaluated these cytokines in blood, but not in muscle. However, we should consider that these are circulating cytokines and this may offer an explanation in regard to muscle inflammatory status. Another limitation is the lack of control subjects of the same age to evaluate cytokine concentrations. The main strength of this study is that we demonstrated, for the first time, differences in inflammatory status among groups with different levels of muscle function and nutritional status. Because inflammation plays a key role in the pathological progress, it is necessary to evaluate other contributors to the inflammatory process during the course of dystrophy such as other cytokines and immunological cells to propose different management procedures in these patients.

## 5. Conclusions

To our knowledge, these novel results present evidence regarding inflammatory status in DMD patients with different levels of muscle function. In the first state of the pathology when patients have better muscular function, the systemic inflammation status is increased. This inflammation is decreased in patients with poorer muscle function. Concentration of systemic inflammatory molecules is independent of nutritional status.

## Figures and Tables

**Table 1:** Clinical, anthropometric, and biochemical parameters in DMD patients.

DMD patients	
*n* = 66	

Age (years)	9.4 ± 3.1
Wheelchair bound (number of boys)	18
Vignos Scale	3.68 ± 2.74
Body weight (kg)	28.8 ± 13.0
Height (m)	1.3 ± 0.2
Body fat mass (%)	28.1 ± 14.2
Body fat mass (kg)	9.1 ± 8.7
Lean body mass (kg)	18.3 ± 5.2
Percentile of BMI	46.3 ± 35.6
IL-6 (pg/mL)^*^	3.2 ± 1.3
IL-1 (pg/mL)^**^	6.04 ± 1.33
TNF–*α* (pg/mL)^***^	10.7 ± 2.8
Adiponectin (ng/mL)	34.14 ± 19.57
Leptin (ng/mL)	9.74 ± 9.15
CRP (mg/dL)	1.9 ± 1.9
CK (U/L)	11475.0 ± 6830.5

DMD: Duchenne muscular dystrophy; IL-6: cytokine 6; IL-1: cytokine 1; BMI: body mass index; TNF-*α*: tumor necrosis factor; CRP: C-reactive protein; CK: creatine kinase. Data are expressed as mean ± SD. Values reported in healthy subjects of ^*^IL–6, 1.93 ± 1.38 pg/mL; ^**^IL-1, 3.6 ± 1.0; ^***^TNF–*α* (pg/mL) 3.6 ± 0.9 pg/mL [7, 17, 18].

**Table 2:** Inflammatory parameters and body composition in DMD patients with different levels of muscle function.

	Vignos Scale A	Vignos Scale B	Vignos Scale C
	(1–5)	(6–8)	(9-10)
	*n* = 44	*n* = 6	*n* = 8
Age (years)	8.04 ± 2.08^a^	11.04 ± 3.49^b^	13.43 ± 1.70^b^
IL-6 (pg/mL)	2.99 (2.00, 9.01)^a^	3.27 (2.00, 4.23)^a^	2.46 (2.00, 4.37)^a^
IL-1 (pg/mL)	6.18 (0.23, 28.30)^a^	8.46 (7.76, 10.30)^b^	1.44 (1.22, 2.82)^c^
TNF-*α* (pg/mL)	11.24 ± 2.91^a^	10.53 ± 1.86^a,b^	8.71 ± 2.17^b,c^
Adiponectin (ng/mL)	33.72 ± 20.40^a^	51.83 ± 33.04^a^	42.62 ± 15.19^a^
Leptin (ng/mL)	7.59 ± 7.86^a^	15.00 ± 6.66^b^	23.10 ± 18.65^b^
CRP (mg/dL)	0.72 (0.30, 7.44)^a^	1.31 (0.30, 5.46)^a^	3.94 (2.52, 6.96)^b^
CK (U/L)	14332.14 ± 6382.63^a^	6241.66 ± 3090.21^b^	3987.50 ± 1818.74^b,c^
Body weight (kg)	24.24 ± 7.99^a^	35.50 ± 13.00^b^	48.00 ± 18.10^b^
Height (m)	1.20 ± 0.11^a^	1.34 ± 0.16^b^	1.52 ± 0.16^b^
Body fat mass (%)	23.25 ± 11.09^a^	41.88 ± 8.57^b^	49.93 ± 6.88^c^
Lean body mass (kg)	16.90 ± 3.70^a^	18.78 ± 4.40^a,b^	22.44 ± 6.15^b^
Body fat mass (kg)	6.00 ± 4.74^a^	14.80 ± 8.56^b^	24.10 ± 11.91^c^

DMD: Duchenne muscular dystrophy; IL-6: cytokine 6; IL-1: cytokine 1; TNF-*α*: tumor necrosis factor; CRP: C-reactive protein; CK: creatine kinase.

The parameter of Vignos Scale was grouped according to Vignos scale A (0–5 points), Vignos Scale B (6–8 points), and Vignos Scale C (9-10 points).

Values are presented as mean ± SD.

Different superscript letters (a, b, and c) indicate statistical significance. *P* < 0.05 (ANOVA, comparison among Vignos Scale groups using Bonferroni post hoc test). Some columns show two letters indicative of statistical significance (no difference or difference with other groups).

**Table 3:** Anthropometric, clinical, and inflammatory parameters in DMD patients with different levels of nutritional status.

	Underweight	Healthy weight	Overweight/obese
	*n* = 12	*n* = 39	*n* = 15
Age (years)	9.31 ± 3.34^a^	9.32 ± 3.19^a^	9.69 ± 2.82^a^
Body weight (kg)	19.51 ± 4.87^a^	27.32 ± 10.60^b^	40.03 ± 15.74^c^
Height (m)	1.22 ± 0.18^a^	1.27 ± 0.17^a^	1.32 ± 0.16^a^
Body fat mass (%)	14.89 ± 5.34^a^	26.87 ± 13.29^b^	41.92 ± 8.29^c^
Lean mass (kg)	15.63 ± 3.85^a^	17.91 ± 4.11^ab^	21.53 ± 7.04^bc^
Body fat mass (kg)	2.62 (0.88, 5.42)^a^	6.30 (1.58, 30.47)^b^	13.14 (8.85, 45.08)^c^
Leptin (ng/mL)	3.00 ± 1.40^ab^	7.13 ± 6.44^ab^	14.99 ± 10.11^c^
Adiponectin (ng/mL)	48.17 ± 26.85^a^	39.63 ± 20.84^a^	30.36 ± 19.46^a^
IL-6 (pg/mL)	2.71 (2.00, 4.21)^a^	3.09 (2.00, 9.00)^a^	3.40 (2.00, 4.12)^a^
IL-1 (pg/mL)	7.20 (0.34, 14.00)^a^	4.60 (0.23, 28.50)^a^	7.62 (1.54, 10.30)^a^
TNF-*α* (pg/mL)	11.12 ± 2.74^a^	10.76 ± 3.15^a^	10.19 ± 1.97^a^
CRP (mg/dL)	0.47 (0.30, 1.98)^a^	1.01 (0.30, 6.96)^b^	2.97 (0.30, 7.44)^b^
CK (U/L)	11650.00 ± 6217.75^a^	12389.45 ± 7404.90^a^	9079.33 ± 5494.83^a^
Vignos Scale	3.0 (2.0, 5.0)^a^	2.0 (1.0, 9.0)^a^	4.00 (1.0, 10.0)^a^

DMD: Duchenne muscular dystrophy; IL-6: cytokine 6; IL-1: cytokine 1; TNF-*α*: tumor necrosis factor; CRP: C-reactive protein; CK: creatine kinase. Values are presented as mean ± SD. Different superscript letters (a, b, and c) indicate statistically significant difference. *P* < 0.05 ANOVA, comparison among different levels of nutritional status using Bonferroni's post hoc test. Some columns show two letters indicative of statistical significance (no difference or different from other groups).
